# Effects of salinity on the cellular physiological responses of *Natrinema* sp. J7-2

**DOI:** 10.1371/journal.pone.0184974

**Published:** 2017-09-19

**Authors:** Yunjun Mei, Huan Liu, Shunxi Zhang, Ming Yang, Chun Hu, Jian Zhang, Ping Shen, Xiangdong Chen

**Affiliations:** 1 School of Chemical and Environmental Engineering, Wuhan Polytechnic University, Wuhan, Hubei, China; 2 State Key Laboratory of Virology, College of Life Science, Wuhan University, Wuhan, Hubei, China; Medical University Graz, AUSTRIA

## Abstract

The halophilic archaea (haloarchaea) live in hyersaline environments such as salt lakes, salt ponds and marine salterns. To cope with the salt stress conditions, haloarchaea have developed two fundamentally different strategies: the "salt-in" strategy and the "compatible-solute" strategy. Although investigation of the molecular mechanisms underlying the tolerance to high salt concentrations has made outstanding achievements, experimental study from the aspect of transcription is rare. In the present study, we monitored cellular physiology of *Natrinema* sp. J7-2 cells incubated in different salinity media (15%, 25% and 30% NaCl) from several aspects, such as cellular morphology, growth, global transcriptome and the content of intracellular free amino acids. The results showed that the cells were polymorphic and fragile at a low salt concentration (15% NaCl) but had a long, slender rod shape at high salt concentrations (25% and 30% NaCl). The cells grew best in 25% NaCl, mediocre in 30% NaCl and struggled in 15% NaCl. An RNA-seq analysis revealed differentially expressed genes (DEGs) in various salinity media. A total of 1,148 genes were differentially expressed, consisting of 719 DEGs (348 up-regulated and 371 down-regulated genes) between cells in 15% vs 25% NaCl, and 733 DEGs (521 up-regulated and 212 down-regulated genes) between cells in 25% vs 30% NaCl. Moreover, 304 genes were commonly differentially expressed in both 15% vs 25% and 25% vs30% NaCl. The DEGs were enriched in different KEGG metabolic pathways, such as amino acids, glycerolipid, ribosome, nitrogen, protoporphyrin, porphyrin and porhiniods. The intracellular predominant free amino acids consisted of the glutamate family (Glu, Arg and Pro), aspartate family (Asp) and aromatic amino acids (Phe and Trp), especially Glu and Asp.

## Introduction

The discovery of Archaea as a distinct domain of life occurred in the late 1970s [1–3], since then the research about the unique community has flourished, particularly in the ecology of extremophiles [4]. Archaea are the prokaryotic microbes generally living in extreme environments, including high or low pH, low oxygen content, temperature and high salinity. Among them, the halophilic archaea (haloarchaea, belonging to the Halobacteriaceae family), inhabit saline environments, such as salt lakes, salt ponds, marine salterns, and even hypersaline environments with NaCl concentrations up to saturation [5]. It is well known that cell membranes are permeable to water, cells cannot maintain the water activity of their cytoplasm higher than that of the surroundings, particularly in high ion environments, which would lead to a rapid loss of intracellular water. To cope up with the adverse environments, haloarchaea have to maintain the cytoplasm at least isoosmotic with the extracellular environments [6]. Physiological study of haloarchaea has revealed two fundamentally different strategies by which these microorganisms achieve osmotic equilibrium: (a) the "salt-in" strategy which involves accumulation of equimolar concentrations of inorganic ions in the cytoplasm and (b) the "compatible-solute" strategy which involves the accumulation of highly organic compatible solutes.

Organisms employing the salt-in strategy selectively uptake K^+^ and Cl^-^ ions inside the cytosol so as to maintain the ionic concentration in the cell equivalent or higher than the external environment [7]. These organisms have a predominance of acidic charged proteome with proteins containing most of their negative charges on their surface [8]. The compatible-solute strategy is broadly known in Domain Archaea, Bacteria, as well as Eukarya. Organisms accumulate organic solutes by uptake from environments or *de novo* synthesize organic compounds like sugars and polyols, amino acids and their derivatives, and compatible solutes for protection against salinity stress [9–15]. However, *Halobacterium* sp. NRC-1 was reported that it not only accumulated potassium ion in cytoplasm under osmotic stress, but also accumulated other compatible solutes [16]. In addition, further studies have also displayed that the lipid composition and protein glycosylationof haloarchaea were affected by the salinity in their natural settings [4, 17,18].

*Halobacterium* sp. NRC-1, a model of halophilic archaea, was extensively investigated the physiological responses to salt stress from the biochemical and molecular aspects [8, 16,19–22]. The results showed that growth under salt stress conditions resulted in modulation of genes coding for ion transporters (including potassium, phosphate, iron transporters, as well as some peptide transporters and stress proteins) [19] and accumulation of compatible solutes (glycine betaine and glutamate) [16]. Although insights into the mechanisms of haloarchaeal adaptation to salt stress have been obtained, there have been few studies regarding transcriptional profiling of salt tolerance mechanisms in extremely halophilic archaea [5, 19, 23, 24]. In the present study, we conducted a comprehensive physiological detection in *Natrinema* sp. J7-2 under salt stress conditions to reveal the molecular mechanisms related to the halo-adaptation of extreme haloarcheae. *Natrinema* sp. J7-2 is an extremely halophilic archaeon isolated from a salt mine in China [25]. Its genome was sequenced, and the whole genome was composed of a 3,697,626-bp chromosome and a 95,989-bp plasmid pJ7-1 [26].

## Materials and methods

### Strain, media, and growth conditions

*Natrinema* sp. J7-2 cells were grown aerobically at 37°C in different media (180 r/min). With the exception of the NaCl concentration, all media contained the same components, including (per liter) 30 g of MgCl_2_.6H_2_O, 2 g of Bacto yeast extract (Difco laboratories, Detroit, USA), 2.5 g of lactalbumin hydrolysate (Difco laboratories, Detroit, USA), and 80 ml of 1 mol/L Tris-HCl (pH 7.2). The media contained various amounts of NaCl (per liter) 150 g, 250 g, or 300 g, which were designated as a percentage of the corresponding medium, e.g., 15%, 25% and 30% NaCl. Cells incubated in 15%, 25% and 30% NaCl were designated as Nat_15, Nat_25 and Nat_30, respectively.

### Sample collection and estimation of colony forming units

*Natrinema* sp. J7-2 cells were incubated in 15%, 25% and 30% NaCl for 26 h, they were in early, middle and early logarithmic phase, respectively. Eight equivalent samples from each medium were collected and centrifuged (13,523 × g for 6 min; Eppendorf 5424R, Hamburg, Germany). The supernatant was removed, and the pellets were collected for subsequent experiments or immediately frozen at -80°C until use. Two equivalent samples were used for electron microscopy, and the remaining six samples were divided into two groups, one for RNA extraction and the other for an amino acid assay. At the same time, the colony forming units (CFU) per ml of cells in different salinity were determined using a dilution plate method.

### Electron microscopy

Samples for thin-section electron microscopy (HITACHI-HT 7700, Hitachi High-Tech, Tokyo, Japan) were prepared according to a previously described method [27]. Samples for scanning electron microscopy (HITACHI S-3000N, Hitachi High-Tech, Tokyo, Japan) were done according to the previous protocol with modification [28], instead of 2.5% glutaraldehyde in 0.2 mol/L phosphate buffer using 2.5% glutaraldehyde in NaCl solution with the NaCl concentration identical to that of the corresponding media during the process of immobilization.

### RNA extraction and quality assessment

The RNeasy^®^ plus Mini Kit with genomic DNA eliminator columns (Qiagen GmbH, Hilden, Germany) was used to extract the total RNA. RNA contamination (contaminated by genomic DNA) and degradation was analyzed on 1% agarose gels. The purity, concentration, and integrity of the RNA were assessed with a Nano Photometer^®^ spectrophotometer (Implen, CA, USA), a Qubit^®^ RNA Assay Kit using a Qubit^®^ 2.0 Fluorometer (Life Technologies, CA, USA) and a Nano 6000 Assay Kit with the Bioanalyzer 2100 system (Agilent Technologies, CA, USA), respectively.

### Library preparation for strand-specific transcriptome sequencing

The MICROBExpress^™^ Bacterial mRNA Enrichment Kit (Ambion, USA) was used to enrich the mRNA from the total RNA according to the operating manual. A total amount of 3 μg purified RNA per sample was used to construct library. The protocol for preparing a cDNA library was according to a previously described method [29]. The products were purified (AMPure XP system, Beckman, USA) and the library quality was assessed using an Agilent Bioanalyzer 2100 system (Agilent, USA). The clustering of samples was performed on a cBot Cluster Generation System using a TruSeq PE Cluster Kit v3-cBot-HS (Illumia) according to the manufacturer’s instructions. After cluster generation, the library preparations were sequenced on an Illumina Hiseq 2000 platform, and 100 bp paired-end reads were generated. All sequence data have been deposited in the Short Read Archive at the NCBI database under the projection accession number SRP093096.

### Quality control

Clean reads were obtained by removing the reads containing adapters, poly-N and low quality reads from raw data. At the same time, the contents of Q20, Q30, and GC were calculated. All of the downstream analyses were based on clean, high quality reads. The software Bowtie 2–2.06 was used to match the clean reads with a reference genome (NC_018224.1).

### Differential expression analysis of transcripts

HTSeq v0.5.4p3 was used to count the number of reads mapped to each gene [30]. The RPKM (Reads Per Kilobase of exon model per Million mapped reads) of each gene was calculated based on the length of the gene and reads count mapped to that gene, considering the effect of the sequencing depth and the gene length for the reads count at the same time, and it is currently the most commonly used method for estimating gene expression levels [31]. Prior to the differential gene expression analysis, the read counts for each sequenced library were adjusted by the edgeR program package through one scaling normalized factor. A differential expression analysis of two conditions was performed using the DEGSeq R package [32]. A corrected P-value of 0.005 and log2 (fold change) of 1 were set as the threshold for significantly differential expression.

### KEGG enrichment analysis of DEGs

The Kyoto Encyclopedia of Genes and Genomes (KEGG) is a database resource for understanding high-level functions and utilities of a biological system (http://www.genome.jp/kegg/), the KOBAS software was used to test the statistical enrichment of DEGs in KEGG pathways [33, 34].

### Quantitative real-time RT-PCR assays

A total of six genes that differentially expressed and related to the S-layer and energy metabolism of *Natrinema* sp. J7-2 in the RNA-seq analysis were selected for further validation using real-time RT-PCR. Real-time RT-PCR primers were designed by software Primer Premier 5.0 and synthesized by Invitrogen (Invitrogen, Shanghai) (S1 Table). Each 20μl reaction included 1μl of total RNA, 0.5 μmol L^-1^of each primer, 1.2 μl TakaRa Ex Taq HS Mix, 0.4 μl PrimeScript PLUS RTase Mix, and 10μl of 2× One step SYBR RT-PCR Buffer 4 (Roche). The reactions were subjected to reverse transcription at 42°C for 5 min, and 94°C for 10 s, then followed by 40 cycles each of 94°C for 5 s, 60°C for 25 s. The protocol concluded with a melting curve program using increasing increments of 0.5°C (10 s each; 68°C–99°C). Each gene was quantified relative to the calibrator. Calculations were made using the instrument and equation 2^-ΔΔCt^ [35].

### Amino acid extraction and quantification

Sample pellets were extracted with 70% ethanol (4: 1 ethanol to pellets volume), as previously described [36]. After ethanol removal and the lyophilization of the remaining solution, the sample was resuspended in D_2_O (0.5 ml). If the sample was cloudy, centrifugation to remove any particulate material was required (13,523 × g for 10 min at 4°C; Eppendorf 5424R, Hamburg, Germany). The content of each free amino acid (FAA) was analyzed on a Waters 2690 HPLC (Waters, USA) according to a previously described method [37]. Statistical analysis was performed using the Origin 8.0 software.

## Results and discussion

### Morphologies and growth of incubated in different salinity media

**Fig 1:**
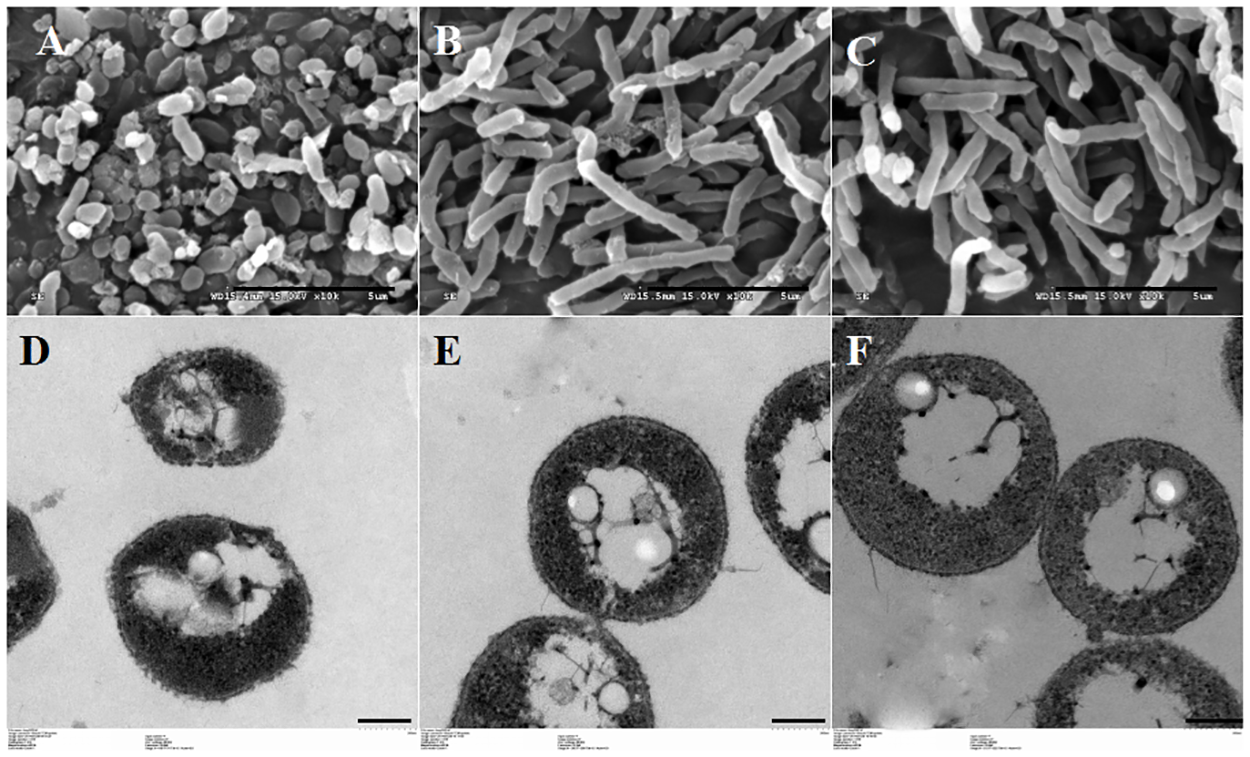
Electron micrographs of *Natrinema* sp. J7-2 cells in media of different salinities. A, B, and C indicate the morphology of cells incubated in 15%, 25% and 30% NaCl media, respectively; the magnification is 10,000-fold; Bar, 5 μm. D, E and F represent the thin-section electron microscopy of the cells cultured in 15%, 25% and 30% NaCl; the magnification is 7,000-fold; Bar, 1μm.

Salinity is a key factor for halophilic organisms that regulates their growth and preserves their cellular structure. *Natrinema* sp. J7-2 cells were incubated in 15%, 25% and 30% NaCl for 26 h, and the cell morphologies was evaluated by scanning electron microscopy; the results are shown in Fig 1. The morphology of the cells incubated in 15% NaCl was polymorphic (Fig 1A), with spherical, irregular and short rod shapes. As far as the shape of the whole cells was concerned, no obvious morphological differences were noted between cells incubated in 25% and 30% NaCl—most cells were long slender rods (Fig 1B and 1C). Our results were similar to the previous report [38] that showed that, in a low enough salt concentration, haloarchaeal cells ceased to grow and the long slender rods assumed irregular and finally spherical shapes before ultimately lysing. Fendrihan et al [39] also reported that the morphology of halophilic archaea was correlated with exposure to different water activity. Thin-section electron microscopy showed that the boundary of cells in 15% NaCl were fuzzy; however, those in 25% and 30% NaCl cellular boundary was clearly evident in 25% and 30% NaCl (Fig 1D, 1E and 1F). These findings suggested that *Natrinema* sp. J7-2 cells in 15% NaCl were more fragile than those at high salinity (25% and 30% NaCl) (data shown in S1 Fig). We inferred that the fragility of the cells resulted from the alteration of the cellular envelopes under low salinity. It was previously reported that haloarchaeal surface-layer (S-layer) glycoproteins were post-translationally modified to adapt to natural environmental salinity [18, 40–42], which affects the structure and composition of the S-layer of haloarchaea. We posited that this phenomenon also occured in *Natrinema* sp. J7-2. In addition, we inferred that the membrane lipid of *Natrinema* sp. J7-2 would also be altered when cells were grown in different salinity media and subsequently affected the stability of cells. Previously, Kellermann et al and Dawson et al demonstrated that the glycerolipid composition of haloarchaea changed under stressful conditions such as critical low or high level of Na^+^ concentration [4, 43].

Plate dilution coating was employed to monitor the growth of *Natrinema* sp. J7-2 cells at different salinities. The CFU/ml were 1.013×10^7^ (15% NaCl), 2.18×10^8^ (25% NaCl) and 2.33×10^7^(30% NaCl), which suggested that the salinity affected the growth of the cells.

### Illumina draft reads and quality control

A paired-end sequencing assay was carried out with an Illumina Hiseq 2000 platform, and 100 bp paired-end reads were generated. The average clean bases in the libraries of three samples (cells in 15%, 25% and 30% NaCl) was more than 1.3Gb (the genome of *Natrinema* sp. J7-2 is 3.79361 Mb). Q20 (> 94%) and Q30 data (84%), the GC content (approximately 64%) and the mapping rate of clean bases (>99%) were satisfactory for subsequent analysis. The quality control data and the mapping rate of clean data were shown in S2 and S3 Tables.

### DEGs of *Natrinema* sp. J7-2 cells in different salinity media

**Fig 2:**
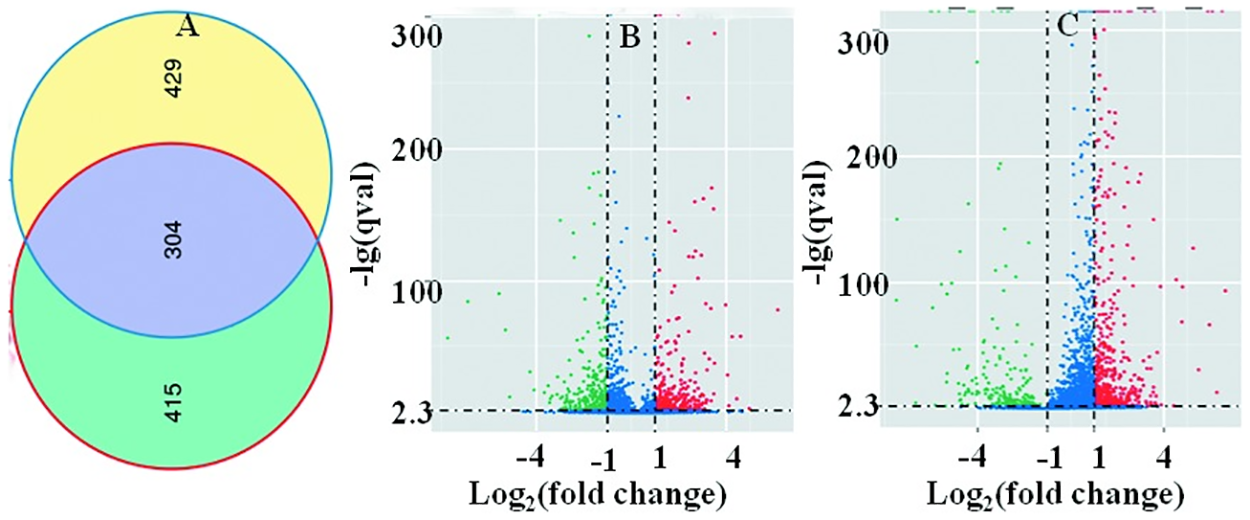
The DEGs of *Natrinema* sp. J7-2 cells in media of different salinities. A, Venn diagram for the comparisons of the DEGs of cells; the red circle represents the DEGs in 15% vs 25% NaCl and the blue circles in 25% vs 30% NaCl. B and C are volcano plots that describe the distribution of the DEGs in 15% vs 25% NaCl and 25% vs 30% NaCl; the red dots are up-regulated genes; the green dots are down-regulated genes; the blue dots indicate genes with no obvious differences.

To better survey the physiological characteristics of *Natrinema* sp. J7-2 cells in different salinities, the DEGs were identified (q value < 0.005 and |log2 (fold change) >1). A comparison of gene expression showed that a total of 1,148 genes were differentially expressed in cells incubated in the three different salinity conditions (Fig 2A), containing 719 (348 up and 371 down) DEGs between cells in 15% vs 25% NaCl, and 733 (521 up and 212 down) between cells in 25% vs 30% NaCl. Moreover, 304 genes were commonly differentially expressed in both 15% vs 25% and 25% vs30% NaCl (Fig 2A). To provide a more intuitive viewpoint, the distribution of DEGs of cells in media of different salinities is shown in Fig 2B and 2C. The volcano plots indicate that the distribution interval of the |log 2 (fold change)| of the DEGs was concentrated between 1 and 4, and only a small part of DEGs were outside of this region. In general, the Venn diagram and volcano plots present the DEGs at the transcript level, which reflects the differences in their physiological and biochemical characteristics.

### KEGG pathway analysis of DEGs

**Fig 3:**
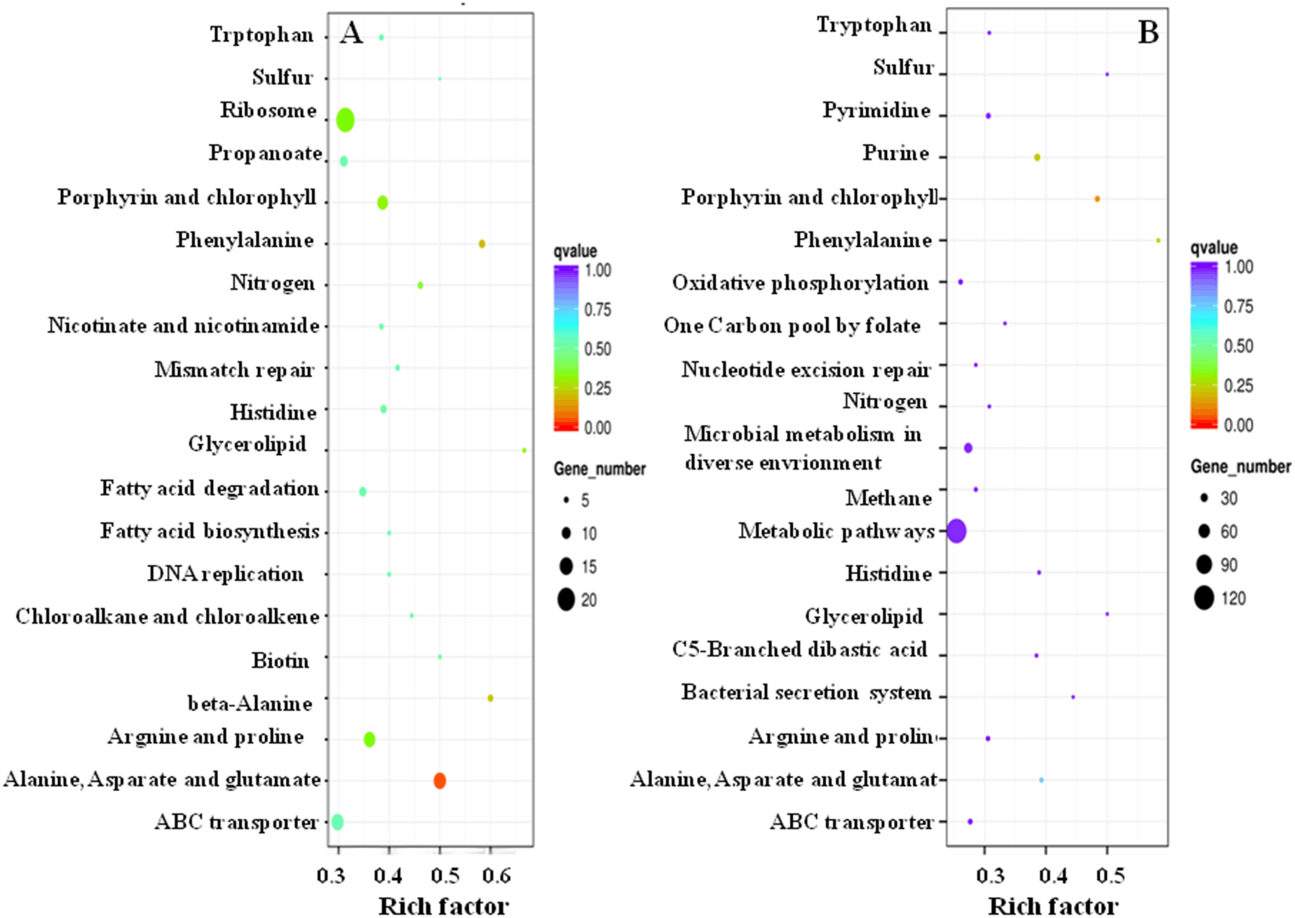
KEGG pathways of DEGs. A, cells in 15% vs 25% NaCl; B, cells in 25% vs 30% NaCl. The rich factor represents the degree of enrichment of DEGs in a pathway; the size of a dot indicates the number of DEGs, the color represents the region of the q-value—the closer to zero, the more highly significant.

To identify the active biochemical pathways of the DEGs, a KEGG pathway analysis is useful for understanding the biological function in media of different salinities. Fig 3 list the top 20 enriched pathways and the most typically enrich pathways (p < 0.05) with their related genes are shown in Table 1. Comparing the enriched pathways in Fig 3A and 3B, half were identical, encompassing amino acids, porphyrin and chlorophyll (function related to protoporphyrin, porphyrins and porphinoids), sulfur, nitrogen, and glycerolipid metabolism and ABC transporters, nutrient and energy metabolism.

**Table 1:** KEGG pathway analysis of DEGs (Nat_15 vs Nat_25).

Metabolism	KEGG_ID/KO*	
	Downregulated genes	Upregulated genes
Ala, Asp and Glu	nat: NJ7G_0685, 1314, 1637, 2607, 3072, 3447, 3448	nat: NJ7G_1211, 1599, 1939, 1940, 1943, 2460, 2673,
Phe		nat: NJ7G_ 1211, 1899, 1908,1914, 1915,1916, 3496
beta-Ala	nat: NJ7G_0204, 1012	nat: NJ7G_ 1141, 1940, 1943, 4348,
Porphyrin and chlorophyll	nat: NJ7G_ 2498	nat: NJ7G_1621, 1624, 1625, 3564, 3565, 3566, 3567, 3568, 3569, 3573, 3582
Glycerolipid	nat: NJ7G_ 0204, 1012	nat: NJ7G_1141, 1713
Arg and Pro	nat: NJ7G_ 0204, 1012, 0348, 0781, 1637, 2613	nat: NJ7G_1141, 1211, 1599, 1939, 1955, 2901, 2902
Ribosome	nat: NJ7G_ 0309, 1154, 1155, 1156, 2050, 2051, 2052, 2053, 2054, 2056, 2057, 2058,2061, 2063, 2070, 2071, 3130, 3133	nat: NJ7G_1674, 3054, 3055
Nitrogen	nat: NJ7G_ 1637, 2607,3007	nat: NJ7G_1599, 1939, 2432

* KEGG ID or KO, the number of DEGs enriched in a KEGG pathway.

According to the degree of enrichment of a DEG per KEGG, the most typically enriched pathways of DEGs of cells incubated in 15% vs 25% NaCl are listed in Table 1 and consist of Ala, Asp and Glu metabolism, Phe metabolism, beta-Alanine metabolism, porphyrin and chlorophyll metabolism, glycerolipid metabolism, Arg and Pro metabolism, ribosome and nitrogen metabolism. The most typically enriched pathways of DEGs in cells incubated in 25% vs 30% NaCl are listed in Table 2 and include porphyrin and chlorophyll metabolism, purine metabolism, Phe metabolism, Ala, Asp and Glu metabolism. Furthermore, amino acid metabolism occupied half of the metabolic pathways identified, including Trp, His, Phe, Arg and Pro, Ala, Asp and Glu metabolism.

**Table 2:** KEGG pathway analysis of DEGs (cells in Nat_25 vs Nat_30).

Metabolism	KEGG_ID/KO	
	Downregulated genes	Upregulated genes
Porphyrin and chlorophyll		nat: NJ7G_ 0370, 0371, 0372, 1114, 2498, 3564, 3565, 3566, 3567, 3568, 3569, 3573, 3579, 3580, 3581
Purine	nat: NJ7G_1537	nat: NJ7G_1093, 1099,1314, 1934, 1568, 2034, 2164, 2239, 2725, 2802, 2916, 3022, 3069, 3123, 3420, 3443, 3822, 3964, 3965, 4041, 4042
Phe	nat: NJ7G_ 1908, 1914, 1915, 1916	nat: NJ7G_0116, 1899, 3496
Ala, Asp and Glu	nat: NJ7G_1599, 2460, 2607	nat: NJ7G_1314, 1503, 2663, 2851, 3123, 3447, 3448, 4042

The above results showed that a marked difference in cellular transcript levels occurs when *Natrinema* sp. J7-2 cells were cultured in different salinities. As for the difference in glycerolipid metabolism (Fig 3A), we think that it plays a role in membrane production and homeostasis of *Natrinema* sp. J7-2, which supports our hypothesis that the saline-dependent fragility of *Natrinema* sp. J7-2 cells resulted from the alteration of membrane lipid.

### Quantitative real-time validation of DEGs

**Fig 4:**
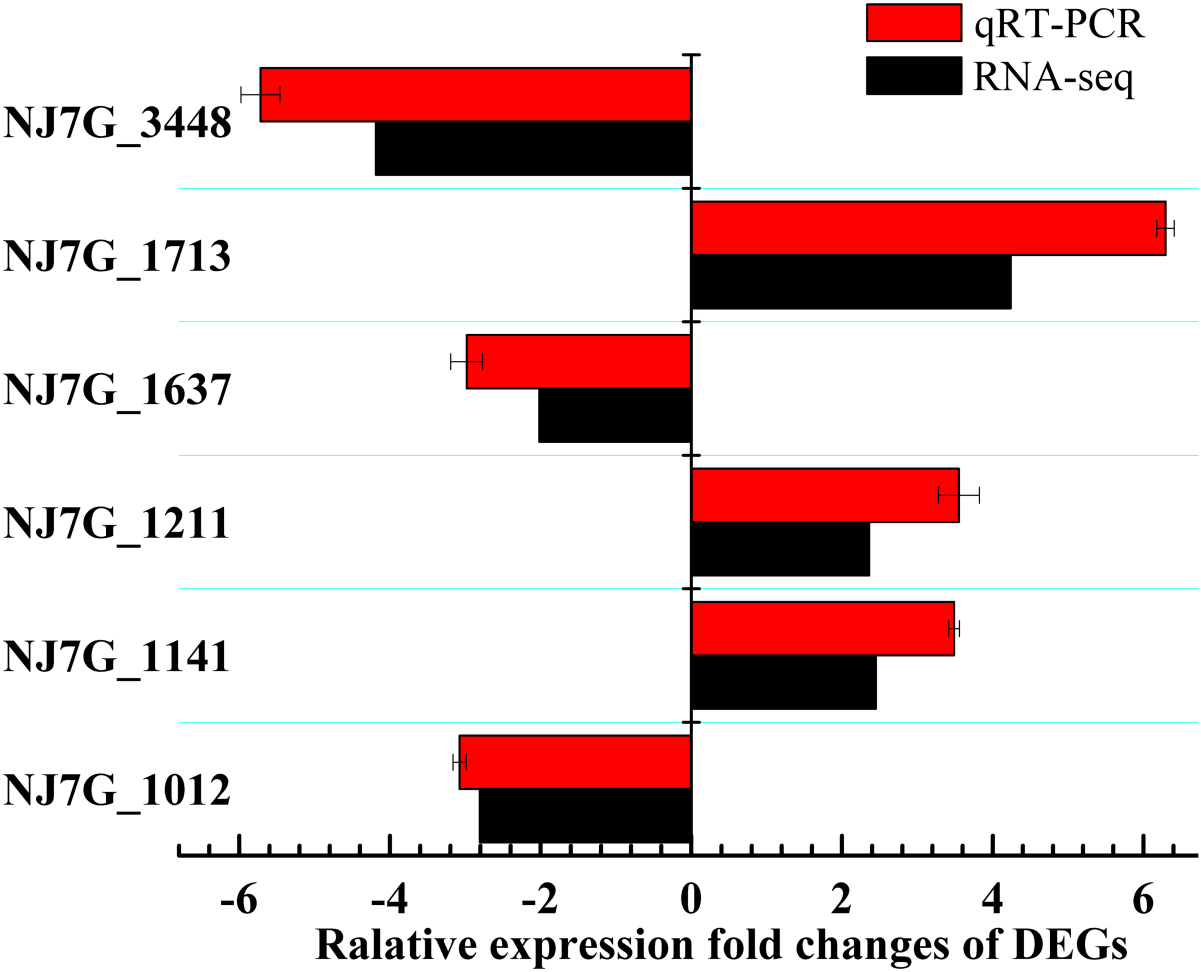
Validation of DEGs by qRT-PCR. Six DEGs riched to the membrane lipid and energy metabolism from RNA-seq were selected to validate by real-time PCR.

To confirm the DEGs in KEGG pathways by RNA-seq, the relative abundances of the selected mRNA were assayed using real-time RT-PCR. Six genes related to the S-layer and energy metabolism of *Natrinema* sp. J7-2 (S1 Table) were selected for further validation. As shown in Fig 4, the genes follow the same trend, which indicated the high quality and reliability of the RNA-seq data analysis, although qRT-PCR is more sensitive, due to the specific primers used for the genes, compared to RNA-seq [44].

### Amino acid analysis

According to the KEGG pathway analysis of DEGs, amino acid metabolisms are the mainly enriched pathway (Fig 3). Under salt stress conditions, amino acids often serve as an osmoprotectant for cells [14,16, 45]. Therefore, we detected the concentration of FAAs related to the amino acid metabolism pathways and the results are shown in Table 3. The content of each FAA was based on 5×10^10^ CFU. Statistically, a significant difference in the FAA concentration of cells cultured in media of different salinities (P < 0.05, from a one-way ANOVA) was indicated, and was identical with the KEGG pathway analysis of DEGs shown above. Taken together, the lowest concentration of each FAA was present in cells incubated in 25% NaCl, not in 15% or 30% NaCl, with the exception of Ala. Glu and Asp were the predominant amino acids of all FAAs, followed by Phe, Arg, Trp, Ala and Pro. Histidine was not detected by HPLC. We speculated that the concentration of His was below the detection limit. Beta-Ala was also not detected because the HPLC method used was unable to distinguish a beta-amino acid from an alpha-amino acid. In general, the result of the amino acids analysis by HPLC is similar to that by RNA-seq.

**Table 3:** Concentration of free amino acids of cells incubated at different salinities.

Amino acid	Amino acid concentration (mg/5×10^10^ CFU)		
	15% NaCl	25% NaCl	30% NaCl
Trp	0.25±0.02	0.011±0.0047	0.11±0.042
Phe	0.64±0.13	0.0023±0	0.09±0
Arg	0.54±0.25	0.071±0.01	0.78±0.14
Pro	0.049±0.02	0.021±0	0.17±0.061
Ala	0.049±0.01	0.03±0	0.23±0.09
Asp	0.64±0.21	0.11±0.06	3.04±0.27
Glu	14.85±3.6	0.39±0.14	3.44±0.35
His	ND*	ND	ND

*ND, Not detectable.

Predominant FAAs consisted of the glutamate family (Glu, Arg and Pro), aspartate family (Asp) and aromatic amino acids (Phe and Trp), especially Glu and Asp, as shown in Table 3. Previously, Lanyi reported halophlic proteins of halophilism showed unique molecular adaptation, including the presence of a large excess of acidic amino acids and small amounts of hydrophobic amino acids [46]; Kokoeva et al reported *Halobacterium* NRC-1accumulated glutamate as compatible solutes under osmotic stress [16].

Organisms employing the "compatible-solute" strategy accumulate organic compatible solutes for protection against salinity stress [9–15], therefore, we deduced that the difference in the content of amino acids of *Natrinema* sp. J7-2 cultured in the different salinity media perhaps was the result of the osmoadaption. Meanwhile, Glu and Asp are major carbon substrates for haloarchaea; they fed into the TCA cycle and subsequently into the respiratory chain for ATP production [20]. Consequently, we suspected that *Natrinema* sp. J7-2 cells accumulated Glu and Asp also as carbon substrates and energy resources when they lived in an adverse environment (in 15% and 30% NaCl media). In addition, acidic amino acids (Glu and Asp) are also an important component of the cell wall to maintain the stability of the haloarchaeal cells [20, 41]. Interestingly, in low-salinity medium, the content of intracellular FAAs of was highest compared with those in moderate and high-salinity media.

Although some interesting results about the physiology of *Natrinema* sp. J7-2 at different salinities were obtained, several problems are still unsolved. In the present study, the RNA-seq data showed that partial DEGs (DEGs of cells cultured in15% NaCl vs 25% NaCl media) was enriched in glycerolipid metabolic pathway, others also reported the component and content of glycerolipids altered with the altering Na^+^ concentration, subsequently affected the membrane motion and permeability [43, 47–51]. However, the relationship between the component and content of glycerolipids of *Natrinema* sp. J7-2 and the salt stress conditions is unclear. *Natrinema* sp. J7-2 is capable of *de novo* synthesis of all amino acids and the biosynthetic pathways have been reconstructed [26]. Our results showed that the total content of intracellular FAAs was different when cells cultured in varying salinity media, but the accumulating pathway of FAAs (by uptake from environment or de novo synthesis) and the role of FAAs (as osmoprotectants, carbon substrates or energy resources) are suspended; unnatural amino acids and derivatives are also not surveyed. All these problems are worthy of attention in the future.

## Supporting information

S1 Table Primers for Real-time RT-PCR analysis. (DOC)

S2 Table The quality control of data. (DOC)

S3 Table The mapping rate of clean data. (DOC)

S1 Fig The statistic results of cellular broken ratio. (DOC)
