# Supplementary material for: Effects of salinity on the cellular physiological responses of Natrinema sp. J7-2
Source: PLoS One. 2017 Sep 19;12(9):e0184974. doi: 10.1371/journal.pone.0184974 (PMC5604999; doi:10.1371/journal.pone.0184974)
Supplement: S1 Table — (DOC) [file pone.0184974.s001.doc]

# S1 Table. Primers for Real-time RT-PCR analysis.

| **Gene** | **Primer sequence(5'-3')** | | **Characteristics** |
| --- | --- | --- | --- |
|  | **Sense** | **Antisense** |  |
| **NJ7G_1713** | AACTGGCTCGTTCTTCCTGA | CGACATATCCTCGAGCCACT | Related to Glycerlipid metabolism, up-regulated |
| NJ7G_1141 | GACAGCCGAAGTGATGTCCT | CGTATCCCGACCACTCAGTT |  |
| NJ7G_1012 | CGATGTCGACTATGGGCTCT | TGTCGTAGACCGTCTTGCTG | Related to Glycerlipid metabolism, down-regulated |
| NJ7G_3448 | GGTAACGACGACCACGAACT | AATGTCCTTGCGTGCAAAC | Related to amino acids metabolism,down-regulated |
| NJ7G_1637 | CGGGACATCATCTACGGACT | AACAGCGAGAGGTGGGTATG |  |
| NJ7G_1211 | **AGAGATGGTCGACACCTTCG** | AATCTCGAGTGCCTCCTTCA | Related to amino acids metabolism, up-regulated |
