# Supplementary material for: Effects of salinity on the cellular physiological responses of Natrinema sp. J7-2
Source: PLoS One. 2017 Sep 19;12(9):e0184974. doi: 10.1371/journal.pone.0184974 (PMC5604999; doi:10.1371/journal.pone.0184974)
Supplement: S2 Table — (DOC) [file pone.0184974.s002.doc]

**S2 Table. The quality control of data.**

| **Sample name*** | **Raw reads** | **Clean reads** | **clean bases** | **Error rate(%)** | **Q20(%)** | **Q30(%)** | **GC content(%)** |
| --- | --- | --- | --- | --- | --- | --- | --- |
| Nat_15_1 | 7533299 | 6855985 | 0.69G | 0.06 | 95.55 | 86.54 | 64.7 |
| Nat_15_2 | 7533299 | 6855985 | 0.69G | 0.07 | 94.34 | 84.05 | 64.22 |
| Nat_25_1 | 6674509 | 6571838 | 0.66G | 0.03 | 96.54 | 92.11 | 64.08 |
| Nat_25_2 | 6674509 | 6571838 | 0.66G | 0.03 | 94.74 | 89.27 | 63.65 |
| Nat_30_1 | 8342011 | 7624577 | 0.76G | 0.06 | 95.77 | 86.81 | 66.66 |
| Nat_30_2 | 8342011 | 7624577 | 0.76G | 0.06 | 95.16 | 85.43 | 65.92 |

* _1, _2, paired-end sequencing assay.
