# Supplementary material for: Effects of salinity on the cellular physiological responses of Natrinema sp. J7-2
Source: PLoS One. 2017 Sep 19;12(9):e0184974. doi: 10.1371/journal.pone.0184974 (PMC5604999; doi:10.1371/journal.pone.0184974)
Supplement: S3 Table — (DOC) [file pone.0184974.s003.doc]

**S3 Table. The mapping rate of clean data.**

| **Sample name** | **Nat_15_1** | **Nat_25_1** | **Nat_30_1** |
| --- | --- | --- | --- |
| Total reads | 13711970 | 13143676 | 15249154 |
| Total mapped | 13601739 (99.2%) | 13034756 (99.17%) | 15110938 (99.09%) |
| Multiple mapped | 250215 (1.82%) | 209603 (1.59%) | 272591 (1.79%) |
| Uniquely mapped | 13351524 (97.37%) | 12825153 (97.58%) | 14838347 (97.31%) |
| Read-1 | 6670883 (48.65%) | 6415221 (48.81%) | 7416072 (48.63%) |
| Read-2 | 6680641 (48.72%) | 6409932 (48.77%) | 7422275 (48.67%) |
| Reads map to '+' | 6678185 (48.7%) | 6414507 (48.8%) | 7416740 (48.64%) |
| Reads map to '-' | 6673339 (48.67%) | 6410646 (48.77%) | 7421607 (48.67%) |
