# Supplementary material for: Effects of salinity on the cellular physiological responses of Natrinema sp. J7-2
Source: PLoS One. 2017 Sep 19;12(9):e0184974. doi: 10.1371/journal.pone.0184974 (PMC5604999; doi:10.1371/journal.pone.0184974)
Supplement: S1 Fig — (DOC) [file pone.0184974.s004.doc]

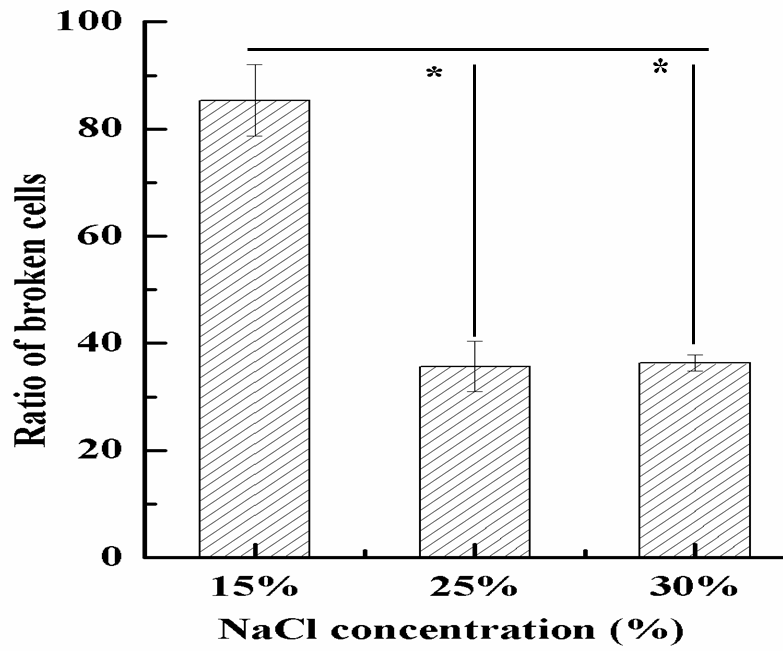


**S1 Fig. The statistic results of cellular broken ratio.** The results showed that the ratio was significantly different compared cells cultured in 15% NaCl with 25% or 30% NaCl. Specifically, the broken ratio of cells cultured in 15% NaCl was much higher than those of in 25% and 30% NaCl; but the broken ratio was very close compared cells in 25% and 30% NaCl . These findings proved that *Natrinema* sp. J7-2 cells in 15% NaCl were more fragile than those at high salinity.
